# Impacts of long-term inorganic and organic fertilization on phosphorus adsorption and desorption characteristics in red paddies in southern China

**DOI:** 10.1371/journal.pone.0246428

**Published:** 2021-01-29

**Authors:** Waqas Ahmed, Huang Jing, Liu Kailou, Sehrish Ali, Han Tianfu, Sun Geng, Chen Jin, Muhammad Qaswar, Du Jiangxue, Sajid Mahmood, Ali Akbar Maitlo, Zulqarnain Haider Khan, Huimin Zhang, Di-Yun Chen

**Affiliations:** 1 National Engineering Laboratory for Improving Quality of Arable Land, Institute of Agricultural Resources and Regional Planning, Chinese Academy of Agricultural Sciences, Beijing, China; 2 Guangdong Provincial Key Laboratory for Radionuclides Pollution Control and Resources, School of Environmental Science and Engineering, Guangzhou University, Guangzhou, China; 3 School of Civil Engineering, Guangzhou University, Guangzhou, China; 4 National Engineering and Technology Research Center for Red Soil Improvement, Jiangxi Institute of Red Soil, Nanchang, China; 5 Hunan Institute of Soil and Fertilizer, Hunan Academy of Agricultural Sciences, Changsha, China; 6 Soil Fertility Research Institute, Agriculture Research Center, Tando Jam, Pakistan; 7 Department of Agriculture, Government of Sindh, Karachi, Pakistan; 8 Agro-Environmental Protection Institute, Ministry of Agriculture of China, Tianjin, China; 9 Chinese Academy of Agricultural Sciences, Beijing, China; Tennessee State University, UNITED STATES

## Abstract

Soil phosphorus (P) adsorption and desorption occur in an important endogenous cycle linked with soil fertility problems and relevant to the environmental risk assessment of P. In our study, the effect of long-term inorganic and organic fertilization on P adsorption and desorption characteristics in relation to changes in soil properties was evaluated by selecting three long-term experimental sites in southern China. The selected treatments at each site were CK (unfertilized), NPK (synthetic nitrogen, phosphorus and potassium) and NPKM (synthetic NPK plus manure). The adsorption and desorption characteristics of P were evaluated using Langmuir and Freundlich isotherms. The results showed that long-term application of NPK plus manure significantly increased soil organic carbon (SOC), total P and available P at all three sites compared with the NPK and CK treatments. All three treatments fit these equations well. The maximum adsorption capacity (Q_m_) of P increased with NPKM treatment, and the binding energy of P (K) and the maximum buffering capacity (MBC) showed increasing trends. NPKM showed the highest Q_m_ (2346.13 mg kg^-1^) at the Jinxian site, followed by Nanchang (221.16 mg kg^-1^) and Ningxiang (2219.36 mg kg^-1^). Compared to CK and NPK, the NPKM treatment showed a higher MBC as 66.64, 46.93 and 44.39 L kg^-1^ at all three sites. The maximum desorption capacity (D_m_) of P in soil was highest with the NPKM treatment (157.58, 166.76, 143.13 mg kg^-1^), showing a better ability to release P in soil. The correlation matrix showed a significant positive correlation of SOC, total and available P with Q_m_, D_m_ and MBC. In conclusion, it is suggested that manure addition is crucial to improve P utilization in red paddy soils within the recommended range to avoid the risk of environmental pollution.

## Introduction

Phosphorus (P) is an important macronutrient required for plant growth and therefore is a vital constituent of fertilizers applied to crops grown worldwide [1]. As the reserves of P are limited, attaining an adequate P supply is a large-scale environmental task in the 21^st^ century [2]. P supply will limit increases in agricultural production worldwide in the coming decades. Excessive usage of fertilizers to overcome P deficiency has led to many problems, such as P accumulation in the soil system and surface water runoff [3, 4]. Red soils, having a low or acidic soil pH, are widely distributed in the southern part of China. Because of the robust adsorption of soil P by aluminum-iron (Al-Fe) oxides, the bioavailability of P to crop plants is inadequate in red soils [5]. In the past few decades, large amounts of organic and inorganic P have been applied to these soils to ensure maximum crop yields. This continuous long-term application of P fertilizer results in the gradual depletion of rock phosphate resources [6]. Moreover, approximately 80% of the applied P is fixed or adsorbed in the soil [7, 8]. The successive movement of P applied as fertilizer results in water eutrophication through runoff [9]. Hence, lessening P adsorption and enhancing the availability of P in red paddy soils is an imperative topic to be focused on in current environmental research.

Adsorption and desorption mechanisms are vital aspects of P behavior with respect to the solid phases in soils. Adsorption processes can limit P phyto-availability, where desorption can allow P to migrate or vanish from soils [9, 10]. P adsorption includes a continuous mechanism of adsorption and precipitation. Adsorption is a reversible but rapid process, whereas the process of precipitation is irreversible and slow, and the difference between these processes is unclear [11, 12]. P desorption, the reverse process of adsorption, has an influential role on soil P availability [13, 14]. It has been stated that the soil P adsorption and desorption process is primarily affected by the soil pH, SOC and Al-Fe oxides [15–17]. Al and Fe oxides are the primary sources of fluctuating charge in red soils. Many studies have reported that SOC results in competitive adsorption by occupying some P adsorption sites [18–20]. In contrast, [21] revealed that SOC can boost the adsorption capability of P in some sandy textured soils.

Previous studies have reported that the adsorption and desorption reactions of soil P directly or indirectly affect P availability and mobility in upland soils [22, 23]. The adsorption and desorption processes of soil P can be analyzed by using several isotherms, i.e., the Langmuir and Freundlich models [24, 25]. The key factors are the adsorption capacity of soil (Q_m_), the adsorption constant (K), the maximum buffer capacity of soil P (MBC), and many others [26]. Continuous fertilizer application can alter soil properties including soil pH, SOC, total P, available P and soil biological indexes [27], which might influence the ability of soil to adsorb P.

To the best of our knowledge, most studies have focused on the adsorption and desorption behavior of P in upland soils with a short experimental duration. However, the impacts of long-term organic and inorganic fertilizer amendment and changing soil properties on soil P adsorption and desorption in red paddy soils have not been well documented. Therefore, it is imperative to explore the effects of fertilization on the phytoavailability of P in these paddy soils. The objectives of this study were to perform batch experiments to examine the adsorption and desorption behavior of P in red paddy soil under long-term organic and inorganic fertilization and the mechanism by which changes in soil properties affect P availability in typical red paddy soils. We hypothesized that the combined application of NPK plus manure would enhance the maximum adsorption capacity of P, P storage capacity, maximum buffering capacity and P binding energy. The study will contribute to an improved understanding of the processes P cycling driven by changes of sorption and adsorption resulted from fertilization practices with intensively managed rice-rice cropping system.

## Materials and methods

### Study sites and sample collection

The three sites under long-term fertilization selected for this study are located in Nanchang (NC) which belongs to Soil, Fertilizer & Resources and Environment Institute, Jiangxi Academy of Agricultural Sciences, Jinxian (JX) and Ningxiang (NX) that belongs to Red Soil Institute of Jiangxi province, China. Research at these experimental sites was carried out with the consent of the Institute of Agricultural Resources and Agricultural regionalization, Chinese Academy of Agricultural Sciences, Beijing, China, under the cooperative research agreement. The geographic locations, climatic details and physicochemical properties of these three sites are given in Table 1.

**Table 1:** Locations, climate conditions and initial surface soil properties of the three long-term experimental sites.

Parameters	Nanchang	Jinxian	Ningxiang
Initiation year	1984	1981	1986
Latitude (N)	28.62	28.71	28.38
Longitude (E)	115.99	116.36	112.72
Climate	MT	SM	SM
Mean annual temperature (°C)	17.8	18.5	16.2
Mean annual precipitation (mm)	1632	1550	1570
Cropping system	Rice-Rice	Rice-Rice	Rice-Rice
Soil classification in FAO	Eutric cambisol	Eutric cambisol	Eutric cambisol
Soil texture	Clay loam	Clay loam	Clay loam
Soil pH	6.08	6.92	6.52
SOM (g kg^-1^)	26.35	29.36	28.54
TN (g kg^-1^)	1.37	1.52	2.05
AN (mg kg^-1^)	82.36	139.66	142.36
TP (g kg^-1^)	0.52	0.53	0.62
AP (mg kg^-1^)	22.36	9.81	13.38
TK (g kg^-1^)	4.26	13.25	21.36
AK (mg kg^-1^)	37.05	83.34	34.48
Fe_d_ (g kg^-1^)	35.66	55.21	32.24
Al_d_ (g kg^-1^)	6.81	10.54	5.46

*Abbreviations: MT: monsoon temperate, SM: subtropical monsoon, R-R: rice-rice, SOM: soil organic matter, TN: total nitrogen, AN: available nitrogen, TP: total phosphorus, AP: available phosphorus, TK: total potassium, AK: available potassium, Fe_d_: dithionite-citrate bicarbonate-extractable Fe and Al_d_: dithionite-citrate bicarbonate-extractable Al.

The agricultural soil of these regions is classified as red soil based on the soil classification system of China [28]. Three treatments were applied in each long-term experiment involving different inorganic and organic fertilization approaches under a rice-based cropping system: (1) CK (unfertilized soil); (2) NPK (synthetic nitrogen, phosphorus and potassium); and (3) NPKM (synthetic NPK plus organic manure). The soil had a heavy clayey texture, containing 38% clay. All the treatments were set in a randomized complete block design (RCBD). Triplicate treatments were separated from each other by cemented ridges to prevent water percolation. Inorganic amendments were applied in the form of urea as nitrogen (N), calcium phosphate as phosphorus (P), and potassium chloride as potassium (K) at each site. The respective annual fertilizer inputs are presented in Table 2.

**Table 2:** Fertilizer input rates (kg ha^−1^) at the three long-term experimental sites.

Sites	Fertilizer application (N-P-K)		
	CK	NPK	NPKM
Nanchang	0-0-0	150-27-125	150-27-125
Jinxian	0-0-0	90-20-63	180-40-125
Ningxiang	0-0-0	143-24-53	143-24-33

For the NPKM treatment, 50% inorganic NPK was applied, and the remaining 50% was added with pig manure based on the N content, while the P and K rates were modified with inorganic fertilizers. Organic manure was applied at rates of 16700 kg ha^-1^, 15000 kg ha^-1^, and 4050 kg ha^-1^ at Nanchang, Jinxian and Ningxiang, respectively. The manure and P fertilizers were applied as a basal dose before rice seedling transplantation, for N and K, 50% was applied as a basal dose, 25% was top-dressed at the tillering stage, and another 25% was added at the panicle stage.

Soil samples were collected from the topsoil (0–20 cm) from all three sites immediately after the early rice harvest using a steel auger. The samples were air-dried, crumbled gently, and passed through a 2 mm sieve before analysis. The representative samples were subjected to analysis for organic C [29], total P [30] and available P [31]. The soil pH was assessed in a 1:2.5 soil and water suspension [32]. The soil properties are given in Table 3.

**Table 3:** The effect of long-term fertilization on soil pH, SOC, total P and available P concentrations and PAC.

Sites	Treatments	pH	SOC (mg kg^-1^)	Total P (g kg^-1^)	AP (mg kg^-1^)	PAC (%)
Nanchang	CK	5.71 ± 0.2 Ab	16.97 ± 0.24 Ca	0.36 ± 0.15 Cb	7.30 ± 0.24 Ca	2.03
	NPK	5.23 ± 0.12 Bb	23.82 ± 0.13 Ba	0.54 ± 0.05 Bb	21.09 ± 1.35 Ba	3.91
	NPKM	5.74 ± 0.15 Ab	28.09 ± 1.33 Aa	1.63 ± 0.07 Ac	82.05 ± 2.14 Ab	5.03
Jinxian	CK	5.62 ± 0.15 ABb	13.25 ± 0.52 Cb	0.49 ± 0.04 Ca	6.51 ± 0.55 Ca	1.33
	NPK	5.50 ± 0.12 Bb	18.87 ± 0.99 Bb	0.93 ± 0.02 Ba	30.14 ± 1.44 Bb	3.24
	NPKM	5.94 ± 0.14 Ab	21.76 ± 1.42 Ab	1.63 ± 0.14 Ab	64.78 ± 1.55 Ac	3.97
Ningxiang	CK	6.34 ± 0.11 Ba	12.11 ± 0.74 Cc	0.45 ± 0.05 Ca	4.61 ± 0.39 Cb	1.02
	NPK	6.51 ± 0.15 Aa	14.35 ± 0.92 Bc	0.96 ± 0.09 Ba	14.82 ± 0.18 Bc	1.54
	NPKM	6.71 ± 0.12 Aa	21.64 ± 1.24 Ab	2.21 ± 0.16 Aa	90.71 ± 2.54 Aa	4.10

*Abbreviations: PAC: phosphorus activation coefficient of soil.

Treatments: CK: unfertilized control; NPK: inorganic nitrogen, phosphorus and potassium; and NPKM: NPK plus manure.

Data (means ± SD, n = 3) followed by different uppercase letters denote significant differences (*P*≤0.05) between fertilization treatments at the same site (A, B, and C), and lowercase letters denote significant differences (*P* ≤ 0.05) between sites for the same fertilization treatment (a, b, and c).

### Zeta potential measurement

Zeta potential is the potential at the shear plane of the electric double layer on colloidal particles. The value and sign (positive or negative) of the zeta potential depend on the surface charges of the soil particles. The isoelectric point (IEP) indicates the pH value at which the zeta potential is 0 mV [33]. The zeta potentials of soil colloids were measured by electrophoresis using a Zetasizer Nano ZS particle size analyzer (ZEN3600, Malvern, Worcestershire, UK). Briefly, 0.05 g of soil sample (0.05 mm) was weighed into a 250-mL Erlenmeyer flask in duplicate followed by the addition of 200 mL of KCl solution (0.01M). Each suspension was dispersed under ultrasound and then dispensed into four 50-mL plastic bottles (~ 30 mL each). The pH was adjusted to 3, 4, 5, 6, or 7 with HCl or KOH. The zeta potential was measured after 2 h of equilibration [34].

### P adsorption and desorption experiments

Adsorption isotherms were assessed using the batch equilibrium method [35]. Briefly, 2.5 g of soil was added to a 50 ml centrifuge tube accompanied by the addition of 25 ml of P working solution, i.e., 0.01 M CaCl_2_. A known concentration of P was maintained: 0 mg L^-1^, 10 mg L^-1^, 20 mg L^-1^, 40 mg L^-1^, 60 mg L^-1^, 100 mg L^-1^ or 150 mg L^-1^. Two to three drops of phenol were further added to inhibit any microbial growth. The mixture was shaken overnight at room temperature (180 r.p.m at 25°C) and then centrifuged (4000g) for approximately 10 minutes. Afterwards, 5 mL of the supernatant was filtered and transferred into a 25 mL tube, and molybdenum blue spectroscopy was used to analyze the P concentration. The soil samples used for adsorption were then washed two times with saturated NaCl solution, centrifuged and subjected to the desorption experiment. After supernatant removal, 25 mL of CaCl_2_ and 3 drops of phenol were added to each sample, followed by overnight shaking. After centrifugation for 10 minutes, 5 mL of the supernatant was taken for the measurement of P concentration by the same molybdenum blue method. This solution P concentration was defined as the desorbed P.

### P adsorption and desorption models

The Langmuir and Freundlich models are widely used for the quantitative description of the P adsorption characteristics of soil particle surfaces [36]. The adsorption isotherms are expressed as:

$$\text{C}/\text{Q}=\text{C}/{\text{Q}}_{\text{m}}+\frac{1}{{\text{K}+\text{Q}}_{\text{m}}}$$ (1)

and

$$\text{Q}={\text{KC}}^{1/\text{n}}$$ (2)

where C is the concentration of P in solution at the equilibrium stage (mg L^-1^); Q is the adsorption capacity (mg kg^-1^); Q_m_ is the maximum P adsorption (mg kg^-1^); K is the equation constant, defined as the factor indicating the intensity of P adsorption, with a maximum quantity consistent with higher soil P adsorption [37]; and n is the constant of heterogeneity related to the intensity of adsorption.

The maximum buffer capacity of P in soil (MBC, mg kg^-1^) was calculated from the series of indexes derived from the Langmuir and Freundlich equations. MBC is an index that merges the P adsorption capacity (Q_m_) with its intensity (K). The resilience of soil to variations in solution P concentration is explained by MBC [38]. This parameter is expressed as:

$$\text{MBC}=\text{K}\times {\text{Q}}_{\text{m}}$$ (3)

### Statistical analysis

All statistical analyses were performed using SPSS 20.0 (IBM SPSS, Somers, NY, USA). Data fitting and mapping were conducted in Origin Pro 19.0 (Origin Lab Corp, MA, USA). Data were subjected to one-way ANOVA for comparison of treatments and two-way ANOVA for comparison of sites, followed by the least significant difference (LSD) test at the *P* ≤ 0.05 level to calculate significant differences among the mean values. Pearson’s correlation coefficients (r) were computed using R 3.6.1 (R Foundation for Statistical Computing, Vienna, Austria). Correlation coefficients were evaluated to determine the relationships between different soil properties, Q_m_, and D_m_.

## Results and discussion

### SOC and total and available P contents in soil

Long-term continuous fertilization significantly (*P* ≤ 0.05) affected the soil chemical properties at all three sites (Table 3). Soil pH showed a nonsignificant trend among the treatments, and it ranged from 5.23 in Nanchang to 6.91 in Ningxiang. Compared with the CK treatments, NPK and NPKM significantly increased the SOC, total P and available P concentrations in the soil. NPKM showed maximum SOC contents of 28.09 mg kg^-1^, 21.76 mg kg^-1^ and 21.64 mg kg^-1^ at Nanchang, Jinxian and Ningxiang, respectively. These results could be due to the manure addition, which supplies enough nutrients to the soil, primarily by directly increasing carbon inputs into the soil, which assists in carbon sequestration. Similar results were reported in previous long-term studies [39, 40]. Long-term NPK plus manure addition significantly increased the soil total P and available P concentrations in the soil at all three sites (Table 3). The maximum values were observed at Ningxiang, with values of 2.21 g kg^-1^ and 90.71 mg kg^-1^, respectively. [41] also reported that long-term fertilization increased soil nutrient concentrations. Similar studies reported that increases in SOC, total P and available P concentrations could be associated with long-term soil mineral and organic inputs that build SOM in the topsoil [42, 43].

The proportion of available P to total P is defined as the P activation coefficient, i.e., PAC, which characterizes the degree of difficulty with which transformation occurs among total P and available P. PAC is a crucial soil fertility indicator, and a higher PAC indicates a maximum P that could be available for plant growth [4, 43]. Similar to SOC, total P and available P, PAC increased significantly with NPKM treatment compared with NPK and CK (Table 3). The PAC values ranged from 1.02 to 5.03 among the treatments at all three sites.

### Characteristics of P adsorption

#### P adsorption isotherms

**Fig 1:**
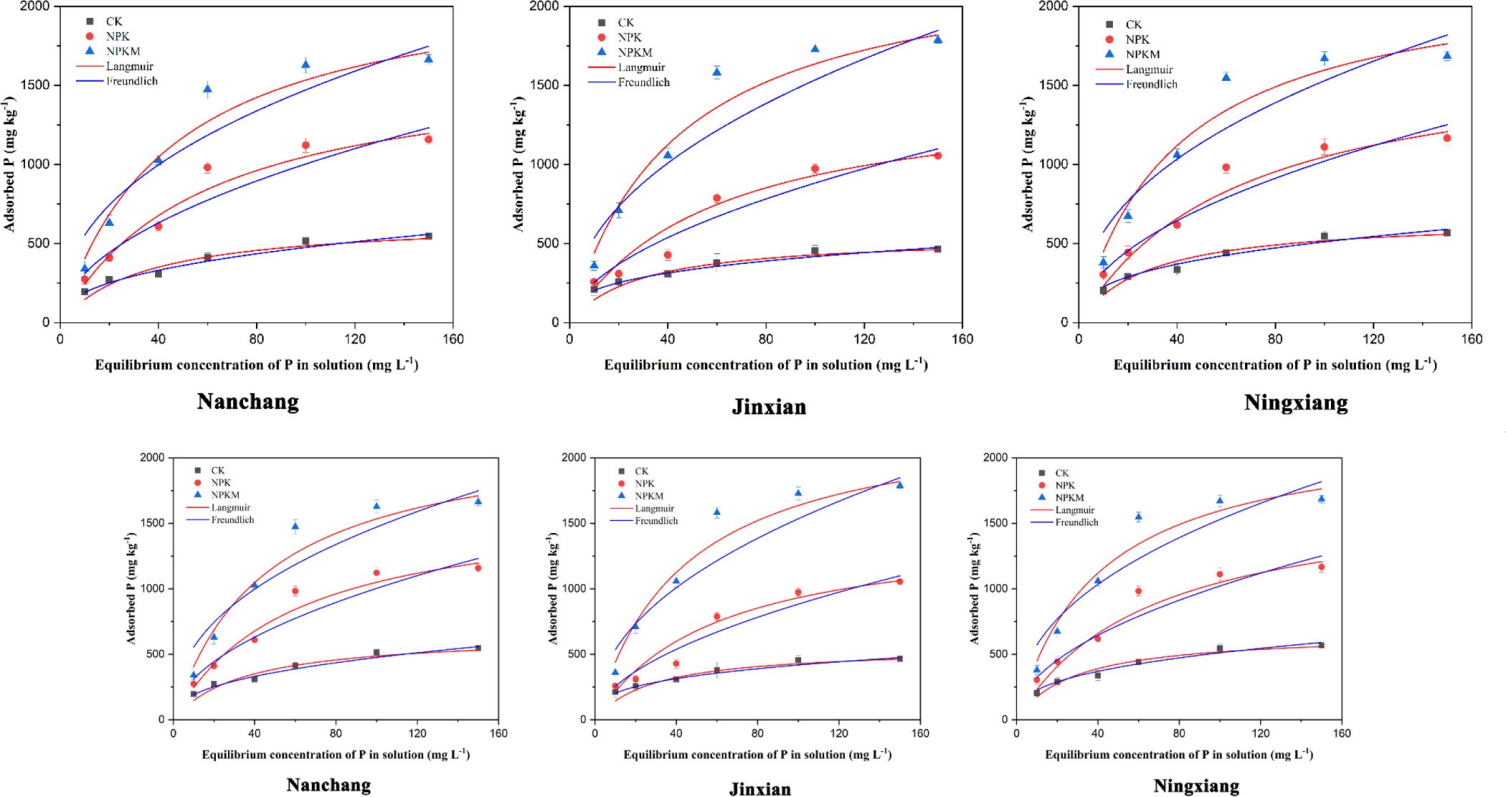
The adsorption isotherms of P in red paddy soils under three different long-term experimental sites.

The sorption isotherms presented in Fig 1 show that the soil samples adsorbed extra P as the exogenous P concentration increased; however, there was a gradual decline in the increasing rate of P adsorption capacity (Fig 1). The variations in the P adsorption capacity between different treatments became increasingly pronounced with increasing added P concentration. Among the treatments, the P adsorption capacity at an added P concentration of 150 mg L^-1^ was maximum for NPKM, with values of 1786.54 mg kg^-1^, 1684.39 mg kg^-1^, and 1663.05 mg kg^-1^ at Jinxian, Ningxiang and Nanchang, respectively. Overall, the P adsorption capacity was higher with NPKM and lower in the CK treatment across all three sites. These results were in line with previous studies [44–46]. This observation was possibly due to the adsorption capacity of the solid phase and the residence time (contact time between the soil and solution) [47]. The P adsorption mechanism comprises two different phases, i.e., chemical and physical adsorption. The process of chemical adsorption dominated at comparatively low exogenous P intensities and was completed rapidly. Ligand and ion exchange are the probable dominant mechanisms that contribute to higher adsorption rates [48]. This phenomenon is commonly termed the relatively quick adsorption phase. At higher P concentrations, the process of chemical adsorption slows rapidly because the existing adsorption sites become saturated so quickly, and the P present in the liquid phase is physicochemically adsorbed to the surface of the soil at a relatively slow rate. This process is termed the slower adsorption phase [44, 48].

Al and Fe oxides are vital for P adsorption, ultimately affecting its availability in soil [49]. SOC alters soil P adsorption by blocking the adsorption sites on Fe/Al oxides or by the formation of Fe/Al-SOC-P multicomplexes with relatively variable structures [50, 51]. Significant differences were found in the P adsorbed amount in soils under different treatments that were subjected to solution with the same exogenous content. The NPKM treatment had the maximum P retention capacity, followed by NPK and CK. This result was probably due to the higher SOC content in NPKM than in the other treatments. These results were also supported by the study of [44].

#### Adsorption equations

Various models have been developed and used for the quantitative description of P adsorption isotherms. The Langmuir and Freundlich models are widely used for P adsorption [10]. The data presented in Table 4 show that the adsorption equations fit the P adsorption isotherms well for different treatments at different sites. The correlation coefficients (R^2^) showed that all the correlations were quite significant. Therefore, either of these equations could be selected to illustrate the characteristics of P adsorption in soils with different treatments. These results were consistent with previous studies [10, 52, 53]. Stated that the P adsorption capacity and its availability are commonly described by the MBC calculated from the Langmuir isotherm, the maximum adsorption capacity (Q_m_), and the binding energy constant (K).

**Table 4:** Isotherm parameters of the Langmuir and Freundlich models for soil P adsorption.

Sites	Treatments	Langmuir equation				Freundlich equation		
		Q_m_ (mg kg^-1^)	K_L_ (L mg^-1^)	R^2^	MBC (L kg^-1^)	n	K_f_	R^2^
Nanchang	CK	653.25	0.02	0.91	13.12	0.39	76.94	0.97
	NPK	1659.26	0.02	0.96	33.19	0.51	97.21	0.93
	NPKM	2221.16	0.03	0.97	66.64	0.42	208.53	0.92
Jinxian	CK	549.15	0.04	0.92	14.97	0.31	98.98	0.97
	NPK	1478.53	0.01	0.97	21.79	0.54	73.1	0.93
	NPKM	2346.13	0.02	0.97	46.93	0.45	186.26	0.92
Ningxiang	CK	649.36	0.02	0.91	12.99	0.39	76.94	0.97
	NPK	1659.26	0.01	0.97	16.61	0.51	97.21	0.94
	NPKM	2219.36	0.02	0.98	44.39	0.39	206.12	0.96

*Abbreviations: Q_m_: maximum P adsorption capacity, K_L_: P adsorption constant, R^2^: model fitting degree, MBC: maximum buffer capacity of soil P.

#### Parameters of P adsorption

The maximum P adsorption capacity (Q_m_) represents the quantity of available adsorption sites per unit soil weight and is commonly used for the determination of soil P adsorption capacity [27, 54]. The Q_m_ values for different treatments across the sites ranged from 549.15 mg kg^-1^ to 2219.36 mg kg^-1^ (Table 4). Long-term inorganic fertilization and manure addition clearly influenced Q_m_. Q_m_ increased with NPKM treatment compared to the other treatments, and the relationships were statistically significant (*P* ≤ 0.05). This difference could be due to increased SOM concentrations because of manure application in the NPKM treatments across the sites, demonstrating that the capacity of soil P storage was enhanced with increased SOC content. Similar results were reported by [55]. In contrast [27], revealed that the SOC content does not directly influence Q_m_. These conflicting findings might have been determined by other soil factors, such as soil pH, soil type, Fe and Al forms, etc., that could have an effect on soil P adsorption capacity. Further studies are recommended to improve the understanding of such influencing factors.

In addition, it has previously been reported that increased SOC content enhances the P adsorption capacity of soils at pH values greater than 6 but decreases the P adsorption capacity at pH values lower than 6 [56]. The P binding energy (K) is important in defining the affinity of soil for P. A maximum K value indicates much stronger adsorption of P [44]. An uneven trend in K values was observed among the treatments (Table 4). This variation could be explained by the SOC content available to react with phosphate anions, as mentioned earlier. MBC is a unified index that merges K and Q_m_ [44]. A high MBC suggests that there will be more P adsorption. The NPKM treatment showed a higher MBC than did the NPK and CK treatments. Similar results were reported in previous studies [57, 58]. This finding led to the assumption that soil P adsorption was mainly controlled by the SOM content in the soil.

### Characteristics of P desorption

#### P desorption isotherms

**Fig 2:**
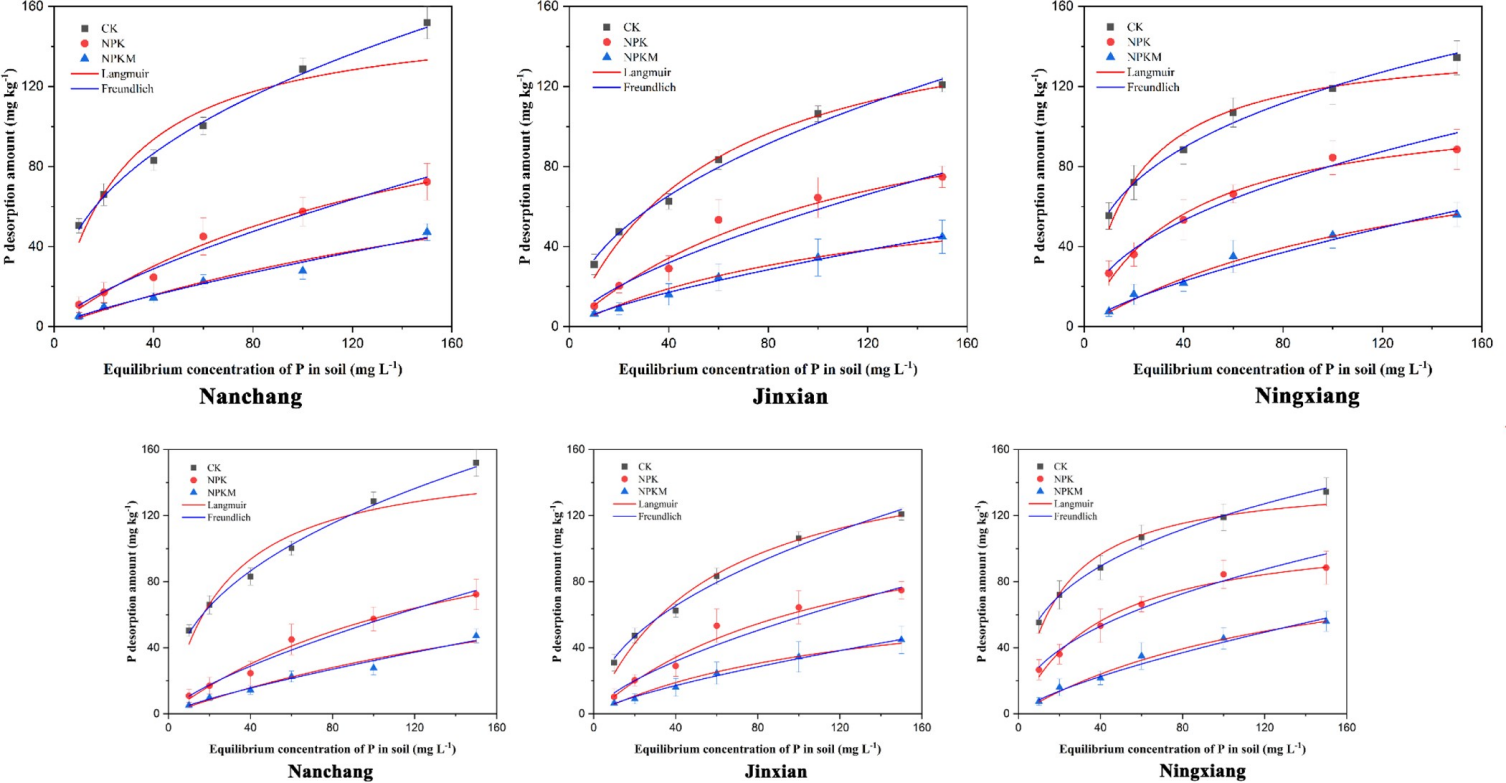
Desorption isotherms of P in red paddy soils at three different long-term experimental sites.

Soil desorption is thought to be an inverse process of sorption. It is considered more important than adsorption due to the significance of immobilized P in soil, which can be reused, and due to environment-related issues that could be caused by P released from soil [4, 44]. The concentration of P desorbed from soil was lower than the amount of P adsorbed in CK, NPK and NPKM (Fig 2). This result indicates that the P adsorbed to the soil could easily be desorbed and be released back into the soil system but that the adsorption was not fully reversible. With increasing exogenous P concentration, the desorbed P amount increased gradually in each treatment, and the trend was more obvious in the case of CK. The rate of P desorption was lower initially when the exogenous P content in solution was lower (< 40 mg L^-1^) but was approximately 20% higher at added P concentrations > 60 mg L^-1^. This result shows that there were abundant adsorption sites on soil colloids when the exogenous P content was lower and there was a higher chemical adsorption binding capacity for soil P, which resulted in a higher adsorption degree and lower desorption. The adsorption sites on soil colloids gradually became covered and saturated as the concentration of exogenous P increased, which resulted in a decreased adsorption binding capacity of the soil. This process is generally described as a physical adsorption level at which physically bound P can be easily desorbed. Consequently, a high P application rate could increase the desorbed P amount.

#### P desorption parameters

The desorption equations fit the isotherms of the CK, NPK and NPKM treatments well, showing significant (*P* ≤ 0.05) correlation coefficients (Table 5). Either the Langmuir or Freundlich equation could be used to describe the desorption characteristics, as was observed in a previous study by Wang and Wang [59]. The amount of P desorbed when the adsorbent was saturated with exogenous P, termed the maximum desorption capacity (D_m_), reflected the maximum amount of P provided to the soil. The D_m_ values ranged from 78.62 mg kg^-1^ to 157.58 mg kg^-1^ across the three sites under CK, NPK and NPKM. The D_m_ values were significantly higher in the NPKM treatment than in the NPK and CK treatments. This difference could be attributed to the higher SOM content in NPKM due to manure addition, showing competition for adsorption sites between SOM and P. This result implies that more SOC entered the soil solution and enhanced the desorbed P amount. However, from an environmental pollution risk assessment perspective, the addition of manure may increase the risk of P loss, primarily by surface water eutrophication. Similar results were previously reported [4].

**Table 5:** P desorption isotherm parameters of the Langmuir and Freundlich models.

Sites	Treatments	Langmuir equation			Freundlich equation		
		D_m_ (mg kg^-1^)	K_L_ (L mg^-1^)	R^2^	n	K_f_	R^2^
Nanchang	CK	124.15	0.003	0.96	0.79	0.83	0.98
	NPK	145.53	0.006	0.98	0.72	2.03	0.98
	NPKM	157.58	0.036	0.92	0.41	18.72	0.99
Jinxian	CK	78.62	0.007	0.97	0.73	1.15	0.99
	NPK	134.13	0.008	0.98	0.66	2.75	0.97
	NPKM	166.76	0.017	0.98	0.48	11.07	0.99
Ningxiang	CK	108.52	0.007	0.99	0.71	1.63	0.98
	NPK	112.58	0.022	0.98	0.45	9.89	0.97
	NPKM	143.13	0.054	0.95	0.32	27.35	0.99

*Abbreviations: D_m_: maximum P desorption capacity, K_L_: P desorption constant, R^2^: model fitting degree.

The results and findings in this study suggest practical implications in soil fertility management, primarily in the use of manure addition to control soil SOM content. These findings will assist in regulating P adsorption and desorption in soil to regulate P nutrition in crop plants. By increasing the SOC content in soil by fertilizer application, the P fixation capacity can be increased. However, when there is a surplus SOC content, sufficient fertilizer must be added to sustain the maximum P supply under long-term experiments.

### Relationships between different soil properties and parameters of P adsorption/desorption

**Fig 3:**
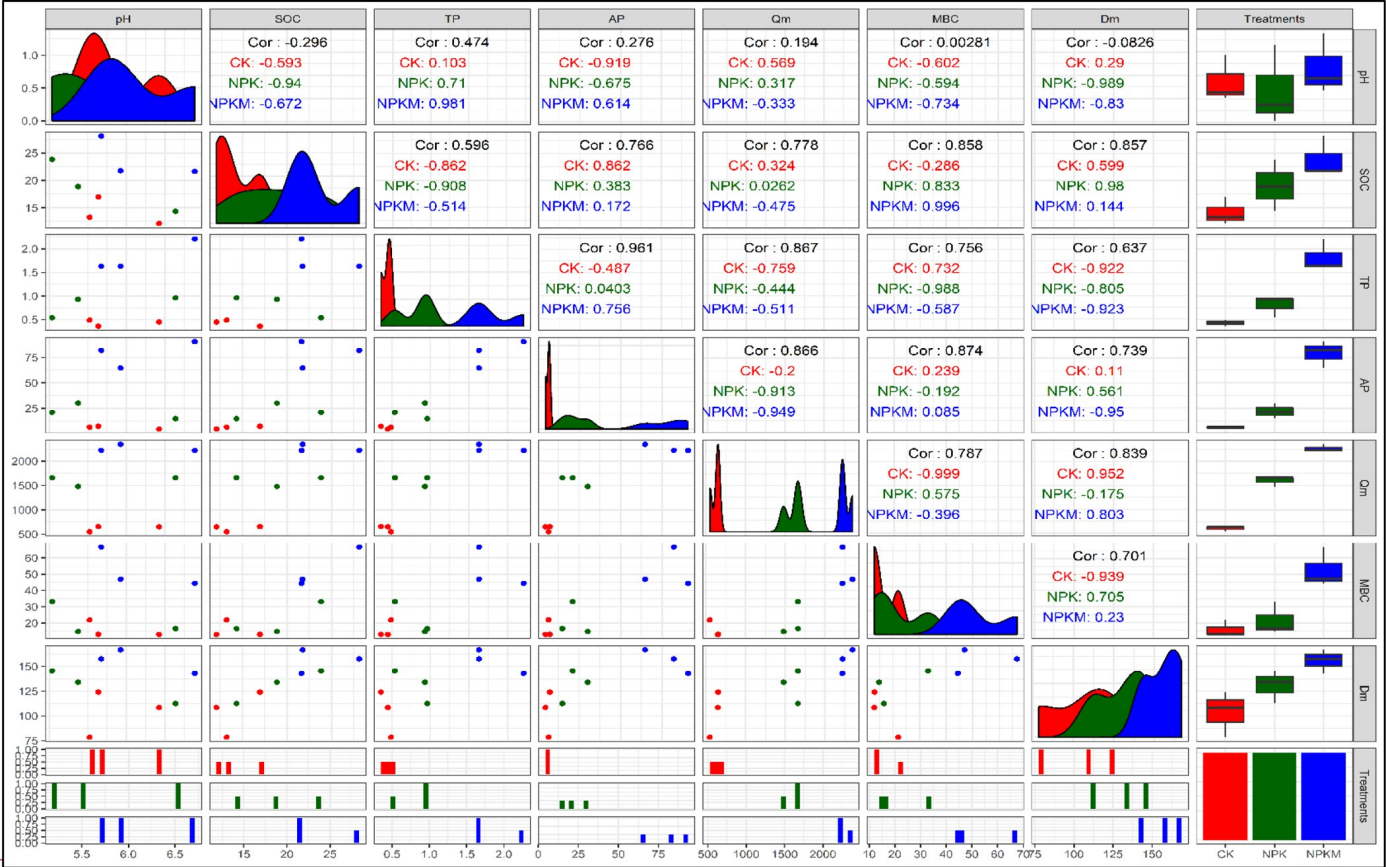
Correlation analysis among soil properties, Q_m_, and D_m_. The upper diagonal represents the correlation coefficient (the overall value is shown in black; the treatments are differentiated by color); the lower diagonal plots show the distribution of data along the axis; the box plots show the overall difference between the treatments.

The correlation matrix showed correlations between soil pH, SOC, total P, available P, Q_m_, MBC and D_m_ (Fig 3). SOC content, total P and available P showed a strong positive correlation with Q_m_, MBC and D_m_.

**Fig 4:**
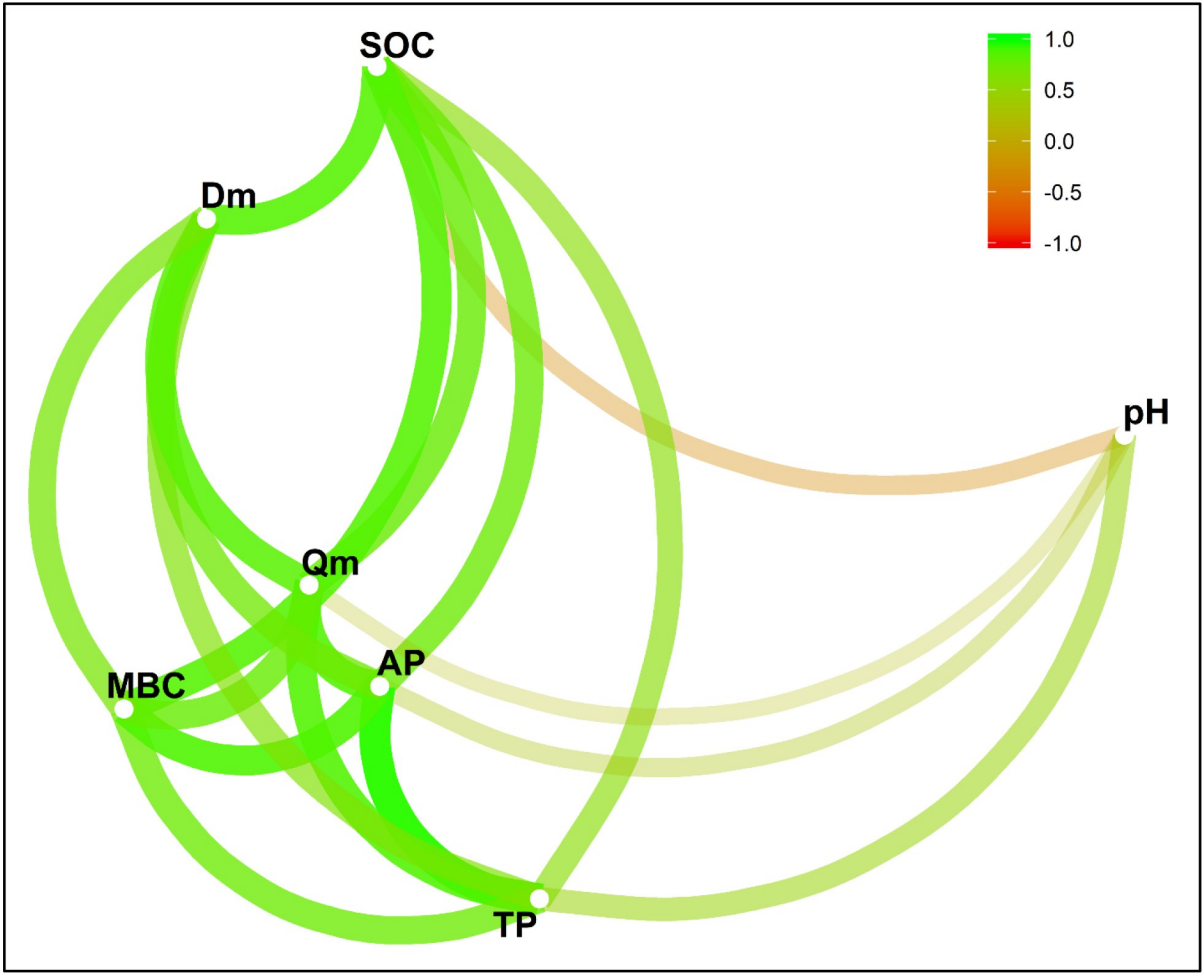
A proposed conceptual framework showing the linkage among soil pH, SOC, total P (TP), available P (AP), Q_m_, D_m_ and the MBC. The linkage is based on the relationships between different variables. The color of each line refers to the degree of relationship between the variables. The variations in the changes in Q_m_ and D_m_ were mainly due to SOC, TP and AP. SOC played a dominant role among these factors. Consequently, adsorption and desorption were affected by the SOC content in the soil.

**Fig 5:**
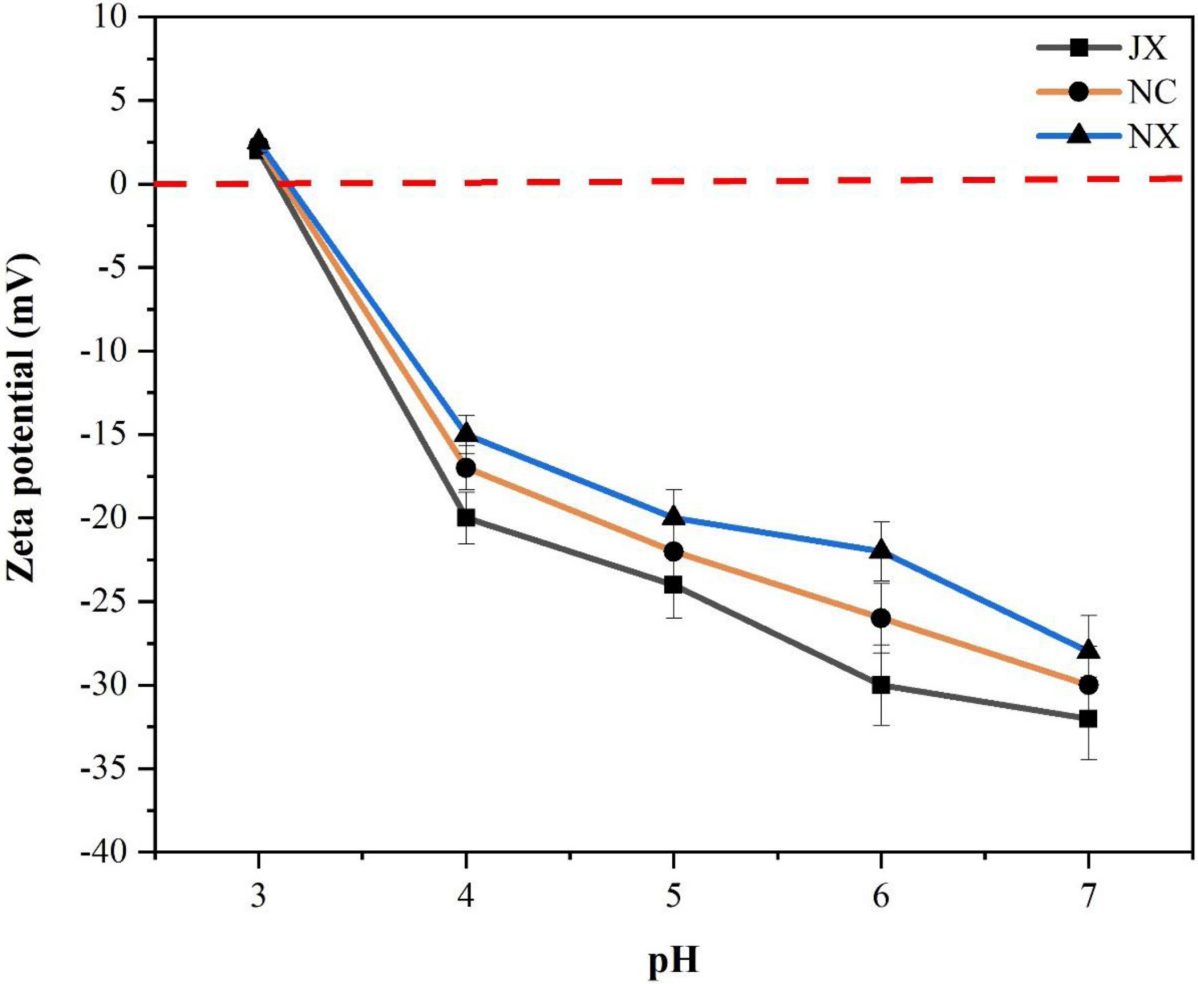
Curves of zeta potential vs. pH for red soils at three different experimental sites.

Organic carbon in soil regulates nutrient supply, and it also interacts with and boosts the supply of other essential nutrients in soil. P adsorption and desorption processes in soil are greatly influenced by SOM content. A similar study was reported by [4]. Fig 4 shows a conceptual framework showing the linkage among soil pH, SOC, total P (TP), available P (AP), Q_m_, D_m_ and MBC. This linkage is based on the relationships between different variables. The variations in the changes in Q_m_ and D_m_ were mainly due to SOC, TP and AP. SOC played a dominant role among these factors. Subsequently, adsorption and desorption were affected by the SOC content in the soil. The absolute value of zeta potential increased with increasing soil pH and ranked in the following order for the three soils: NX< NC <JX (Fig 5). This result indicates that the P adsorption capacity of NX soil was the lowest, while that of JX soil was the highest among the three sites. The high P adsorption capacity of JX soil was confirmed by its high IEP, which indicates that the soil surface was positively charged and advantageous to P adsorption. This is probably due to the naturally acidic parent material which is characteristic of red soils. The weathering of parent material could result in abundant Fe-Al oxides, leading to substantial leaching of soluble soil minerals and basic cations [60].

## Conclusions

The relationships between the soil properties of red paddy soils from southern China under long-term fertilization experiments and their adsorption-desorption characteristics were assessed by different batch experiments. The Langmuir and Freundlich equations fit the isotherms well for every treatment at each site. The combined application of NPK plus manure enhanced the maximum adsorption capacity of P, P storage capacity, maximum buffering capacity and P binding energy. Manure addition significantly altered the MBC and D_m_ values and increased the P desorption process, suggesting an effective supply of P to crops from the soil. In short, it is suggested that manure addition is important to improve P utilization in red paddies within the recommended range to avoid the environmental risk of pollution.

## References

[1] Liu S, Meng J, Jiang L, Yang X, Lan Y, Cheng X, et al Rice husk biochar impacts soil phosphorous availability, phosphatase activities and bacterial community characteristics in three different soil types. Appl Soil Ecol. 2017;116: 12–22. 10.1016/j.apsoil.2017.03.020

[2] Fink JR, Inda A V., Bavaresco J, Barrón V, Torrent J, Bayer C. Adsorption and desorption of phosphorus in subtropical soils as affected by management system and mineralogy. Soil Tillage Res. 2016;155: 62–68. 10.1016/j.still.2015.07.017

[3] van der Salm C, van Middelkoop JC, Ehlert PAI. Changes in soil phosphorus pools of grasslands following 17 yrs of balanced application of manure and fertilizer. Soil Use Manag. 2017;33: 2–12. 10.1111/sum.12333

[4] Yang X, Chen X, Yang X. Effect of organic matter on phosphorus adsorption and desorption in a black soil from Northeast China. Soil Tillage Res. 2019;187: 85–91. 10.1016/j.still.2018.11.016

[5] Messiga AJ, Ziadi N, Angers DA, Morel C, Parent LE. Tillage practices of a clay loam soil affect soil aggregation and associated C and P concentrations. Geoderma. 2011;164: 225–231. 10.1016/j.geoderma.2011.06.014

[6] Gilbert N. The disappearing nutrient: phosphate-based fertilizers have helped spur agricultural gains in the past century, but the world may soon run out of them. Natasha Gilbert. Nature. 2009;461: 716–719. Available: http://go.galegroup.com/ps/i.do?p=AONE&sw=w&issn=00280836&v=2.1&it=r&id=GALE%7CA210224903&sid=googleScholar&linkaccess=fulltext 10.1038/461716a 19812648

[7] Zhu J, Li M, Whelan M. Phosphorus activators contribute to legacy phosphorus availability in agricultural soils: A review. Sci Total Environ. 2018;612: 522–537. 10.1016/j.scitotenv.2017.08.095 28865270

[8] Withers PJA, Edwards AC, Foy RH. Phosphorus cycling in UK agriculture and implications for phosphorus loss from soil. Soil Use Manag. 2006;17: 139–149. 10.1111/j.1475-2743.2001.tb00020.x

[9] Gérard-Marchant P, Hively WD, Steenhuis TS. Distributed hydrological modelling of total dissolved phosphorus transport in an agricultural landscape, part I: Distributed runoff generation. Hydrol Earth Syst Sci. 2006;10: 245–261. 10.5194/hess-10-245-2006

[10] Lair GJ, Zehetner F, Khan ZH, Gerzabek MH. Phosphorus sorption-desorption in alluvial soils of a young weathering sequence at the Danube River. Geoderma. 2009;149: 39–44. 10.1016/j.geoderma.2008.11.011

[11] Arias M, Da Silva-Carballal J, García-Río L, Mejuto J, Núñez A. Retention of phosphorus by iron and aluminum-oxides-coated quartz particles. J Colloid Interface Sci. 2006;295: 65–70. 10.1016/j.jcis.2005.08.001 16125184

[12] Gou X, Cai Y, Wang C, Li B, Zhang Y, Tang X, et al Effects of different long-term cropping systems on phosphorus adsorption and desorption characteristics in red soils. J Soils Sediments. 2020;20: 1371–1382. 10.1007/s11368-019-02493-2

[13] Heidari S, Reyhanitabar A, Oustan S. Kinetics of phosphorus desorption from calcareous soils using DGT technique. Geoderma. 2017;305: 275–280. 10.1016/j.geoderma.2017.06.012

[14] McDonald GJ, Norton SA, Fernandez IJ, Hoppe KM, Dennis J, Amirbahman A. Chemical controls on dissolved phosphorus mobilization in a calcareous agricultural stream during base flow. Sci Total Environ. 2019;660: 876–885. 10.1016/j.scitotenv.2019.01.059 30743973

[15] Quesada CA, Lloyd J, Anderson LO, Fyllas NM, Schwarz M, Czimczik CI. Soils of Amazonia with particular reference to the RAINFOR sites. Biogeosciences. 2011;8: 1415–1440. 10.5194/bg-8-1415-2011

[16] Fang H, Cui Z, He G, Huang L, Chen M. Phosphorus adsorption onto clay minerals and iron oxide with consideration of heterogeneous particle morphology. Sci Total Environ. 2017;605–606: 357–367. 10.1016/j.scitotenv.2017.05.133 28668747

[17] Bornø ML, Müller-Stöver DS, Liu F. Contrasting effects of biochar on phosphorus dynamics and bioavailability in different soil types. Sci Total Environ. 2018;627: 963–974. 10.1016/j.scitotenv.2018.01.283 29426221

[18] Liu Y, Zhu ZQ, He XS, Yang C, Du YQ, Huang YD, et al Mechanisms of rice straw biochar effects on phosphorus sorption characteristics of acid upland red soils. Chemosphere. 2018;207: 267–277. 10.1016/j.chemosphere.2018.05.086 29803158

[19] Zhang Y, Huang S, Guo D, Zhang S, Song X, Yue K, et al Phosphorus adsorption and desorption characteristics of different textural fluvo-aquic soils under long-term fertilization. J Soils Sediments. 2019;19: 1306–1318. 10.1007/s11368-018-2122-0

[20] Ahmed W, Jing H, Kaillou L, Qaswar M, Khan MN, Jin C, et al Changes in phosphorus fractions associated with soil chemical properties under long-term organic and inorganic fertilization in paddy soils of southern China. PLoS One. 2019;14: e0216881 10.1371/journal.pone.0216881 31075143 PMC6510419

[21] Debicka M, Kocowicz A, Weber J, Jamroz E. Organic matter effects on phosphorus sorption in sandy soils. Arch Agron Soil Sci. 2016;62: 840–855. 10.1080/03650340.2015.1083981

[22] Bera R, Seal A, Bhattacharyya P, Mukhopadhyay K, Giri R. Phosphate sorption desorption characteristics of some ferruginous soils of tropical region in Eastern India. Environ Geol. 2006;51: 399–407. 10.1007/s00254-006-0335-9

[23] Ahmed W, Kailou L, Qaswar M, Jing H, Qinghai H, Yongmei X, et al Long-term mineral fertilization improved the grain yield and phosphorus use efficiency by changing soil P fractions in ferralic Cambisol. Agronomy. 2019;9: 784 10.3390/agronomy9120784

[24] Bhattacharyya P, Nayak AK, Shahid M, Tripathi R, Mohanty S, Kumar A, et al Effects of 42-year long-term fertilizer management on soil phosphorus availability, fractionation, adsorption–desorption isotherm and plant uptake in flooded tropical rice. Crop J. 2015;3: 387–395. 10.1016/j.cj.2015.03.009

[25] Guedes RS, Melo LCA, Vergütz L, Rodríguez-Vila A, Covelo EF, Fernandes AR. Adsorption and desorption kinetics and phosphorus hysteresis in highly weathered soil by stirred flow chamber experiments. Soil Tillage Res. 2016;162: 46–54. 10.1016/j.still.2016.04.018

[26] Li RY, Qiu YX, Liu CY, Wang YL, Huang LD. Adsorption-desorption behaviors of phosphorus under different silicon concentrations in paddy soils. Chin J Soil Sci. 2013;44: 1134–1139.

[27] Yan X, Wang D, Zhang H, Zhang G, Wei Z. Organic amendments affect phosphorus sorption characteristics in a paddy soil. Agric Ecosyst Environ. 2013;175: 47–53. 10.1016/j.agee.2013.05.009

[28] Zhang WL, Xu AG, Zhang RL, Ji HJ, Wu SX. Review of soil classification and revision of China soil classification system. Sci Agric Sin. 2014;47: 3214–3230. 10.3864/j.issn.0578-1752.2014.16.009

[29] Walkley A. An examination of methods for determining organic carbon and nitrogen in soils. (with one text-figure). J Agric Sci. 1935;25: 598–609. 10.1017/S0021859600019687

[30] Murphy J, Riley JP. A modified single solution method for the determination of phosphate in natural waters. Anal Chim Acta. 1962;27: 31–36. 10.1016/S0003-2670(00)88444-5

[31] Olsen SR, Cole C V, Watandbe F, Dean L. Estimation of Available Phosphorus in Soil by Extraction with sodium Bicarbonate. Journal of Chemical Information and Modeling. United States Department Of Agriculture; Washington; 1954 10.1017/CBO9781107415324.004

[32] Lu RK. Soil agricultural chemical analysis method. China Agric Sci Technol Press Beijing 2000; 1–315.

[33] Xu RK, Qafoku NP, Van Ranst E, Li JY, Jiang J. Adsorption Properties of Subtropical and Tropical Variable Charge Soils: Implications from Climate Change and Biochar Amendment. Advances in Agronomy. 2016 10.1016/bs.agron.2015.09.001

[34] Jiang J, Xu R, Wang Y, Zhao A. The mechanism of chromate sorption by three variable charge soils. Chemosphere. 2008 10.1016/j.chemosphere.2007.12.012 18291439

[35] Sui Y, Thompson ML. Phosphorus Sorption, Desorption, and Buffering Capacity in a Biosolids-Amended Mollisol. Soil Sci Soc Am J. 2000;64: 164–169. 10.2136/sssaj2000.641164x

[36] Lu H, Yang L, Shabbir S, Wu Y. The adsorption process during inorganic phosphorus removal by cultured periphyton. Environ Sci Pollut Res. 2014;21: 8782–8791. 10.1007/s11356-014-2813-z 24728572

[37] BACHE BW, WILLIAMS EG. a Phosphate Sorption Index for Soils. J Soil Sci. 1971;22: 289–301. 10.1111/j.1365-2389.1971.tb01617.x

[38] Holford ICR. Soil phosphorus: Its measurement, and its uptake by plants. Australian Journal of Soil Research. 1997 pp. 227–239. 10.1071/S96047

[39] Qaswar M, Jing H, Ahmed W, Dongchu L, Shujun L, Ali S, et al Long-term green manure rotations improve soil biochemical properties, yield sustainability and nutrient balances in acidic paddy soil under a rice-based cropping system. Agronomy. 2019;9: 780 10.3390/agronomy9120780

[40] Cai A, Zhang W, Xu M, Wang B, Wen S, Shah SAA. Soil fertility and crop yield after manure addition to acidic soils in South China. Nutr Cycl Agroecosystems. 2018;111: 61–72. 10.1007/s10705-018-9918-6

[41] Lan ZM, Lin XJ, Wang F, Zhang H, Chen CR. Phosphorus availability and rice grain yield in a paddy soil in response to long-term fertilization. Biol Fertil Soils. 2012;48: 579–588. 10.1007/s00374-011-0650-5

[42] Fageria NK, Dos Santos AB, Moraes MF. Influence of urea and ammonium sulfate on soil acidity indices in lowland rice production. Commun Soil Sci Plant Anal. 2010;41: 1565–1575. 10.1080/00103624.2010.485237

[43] Wu Q, Zhang S, Zhu P, Huang S, Wang B, Zhao LP, et al Characterizing differences in the phosphorus activation coefficient of three typical cropland soils and the influencing factors under longterm fertilization. PLoS One. 2017;12 10.1371/journal.pone.0176437 28467425 PMC5415111

[44] Wang L, Liang T. Effects of exogenous rare earth elements on phosphorus adsorption and desorption in different types of soils. Chemosphere. 2014;103: 148–155. 10.1016/j.chemosphere.2013.11.050 24342358

[45] Siddique MT, Robinson JS. Differences in Phosphorus Retention and Release in Soils Amended with Animal Manures and Sewage Sludge. Soil Sci Soc Am J. 2004;68: 1421–1428. 10.2136/sssaj2004.1421

[46] Abdala DB, Ghosh AK, da Silva IR, de Novais RF, Alvarez Venegas VH. Phosphorus saturation of a tropical soil and related P leaching caused by poultry litter addition. Agric Ecosyst Environ. 2012;162: 15–23. 10.1016/j.agee.2012.08.004

[47] Bhadha JH, Daroub SH, Lang TA. Effect of kinetic control, soil: Solution ratio, electrolyte cation, and others, on equilibrium phosphorus concentration. Geoderma. 2012;173–174: 209–214. 10.1016/j.geoderma.2011.12.027

[48] Lai DYF, Lam KC. Phosphorus sorption by sediments in a subtropical constructed wetland receiving stormwater runoff. Ecol Eng. 2009;35: 735–743. 10.1016/j.ecoleng.2008.11.009

[49] Gérard F. Clay minerals, iron/aluminum oxides, and their contribution to phosphate sorption in soils—A myth revisited. Geoderma. 2016;262: 213–226. 10.1016/j.geoderma.2015.08.036

[50] Wang Y, Zhang Y, He Y. Effect of soil matrix components on phosphate sorption index in red soil. Acta Ped0L0Gica Sin. 2012;49: 552–559.

[51] Moshi AO, Wild A, Greenland DJ. Effect of organic matter on the charge and phosphate adsorption characteristics of kikuyu red clay from kenya. Geoderma. 1974;11: 275–285. 10.1016/0016-7061(74)90054-8

[52] Lin J, Zhang Z, Zhan Y. Effect of humic acid preloading on phosphate adsorption onto zirconium-modified zeolite. Environ Sci Pollut Res. 2017;24: 12195–12211. 10.1007/s11356-017-8873-0 28353102

[53] Yan J, Jiang T, Yao Y, Lu S, Wang Q, Wei S. Preliminary investigation of phosphorus adsorption onto two types of iron oxide-organic matter complexes. J Environ Sci (China). 2016;42: 152–162. 10.1016/j.jes.2015.08.008 27090706

[54] Yokoyama D, Mori T, Wagai R, Hiradate S, Kitayama K. Characteristics of phosphorus fractions in the soils derived from sedimentary and serpentinite rocks in lowland tropical rain forests, Borneo. Soil Sci Plant Nutr. 2018;64: 218–221. 10.1080/00380768.2017.1421018

[55] Zhou Q, Zhu Y. Potential pollution and recommended critical levels of phosphorus in paddy soils of the southern Lake Tai area, China. Geoderma. 2003;115: 45–54. 10.1016/S0016-7061(03)00074-0

[56] Zhong X, Zhao X, Bao H, Li H, Li G, Lin Q. The evaluation of phosphorus leaching risk of 23 Chinese soils Ⅰ. Leaching criterion. Acta Ecol Sin. 2004;24: 2275–2280.

[57] WILLIAMS EG, SAUNDERS WMH. Distribution of Phosphorus in Profiles and Particle‐Size Fractions of Some Scottish Soils. J Soil Sci. 1956;7: 90–109. 10.1111/j.1365-2389.1956.tb00866.x

[58] Wang JB, Chen ZH, Chen LJ, Zhu AN, Wu ZJ. Surface soil phosphorus and phosphatase activities affected by tillage and crop residue input amounts. Plant, Soil Environ. 2011;57: 251–257. 10.17221/437/2010-pse

[59] Wang S, Wang E, others. Desorption characteristics of phosphorus from different used sandy soil in Western Liao River Basin. Res Environ Sci. 2011;24: 756–762.

[60] Tellen VA, Yerima BPK. Effects of land use change on soil physicochemical properties in selected areas in the North West region of Cameroon. Environ Syst Res. 2018 10.1186/s40068-018-0106-0

