# Correction: Impacts of long-term inorganic and organic fertilization on phosphorus adsorption and desorption characteristics in red paddies in southern China

**DOI:** 10.1371/journal.pone.0257122

**Published:** 2021-09-01

**Authors:** The PLOS ONE Staff

Figs 1 and 2 are incorrect. The authors have provided corrected versions here.

**Fig 1:**
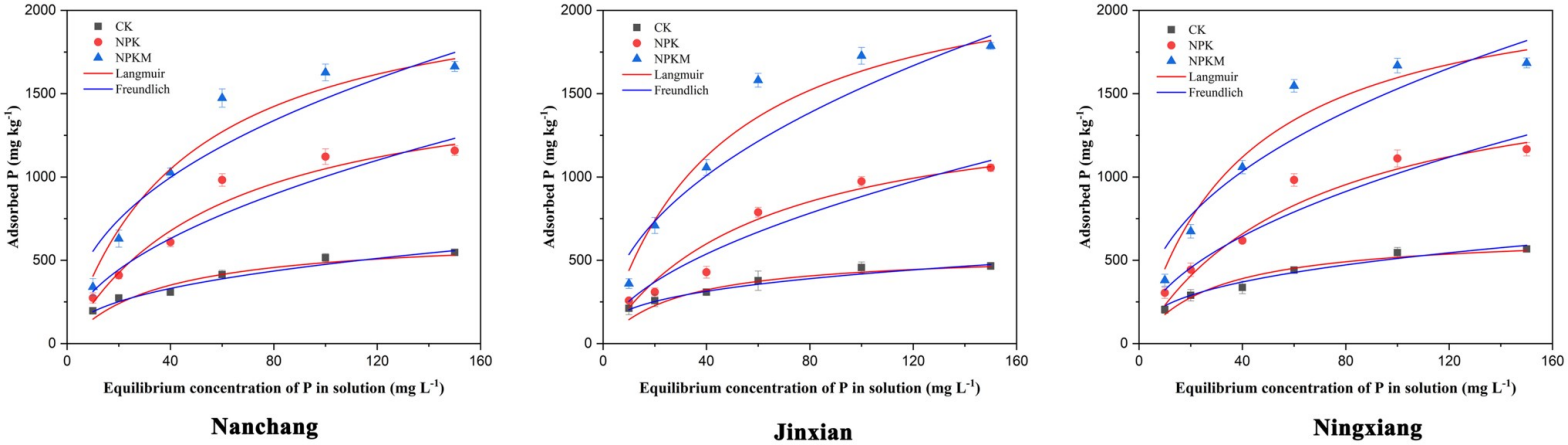
The adsorption isotherms of P in red paddy soils under three different long-term experimental sites.

**Fig 2:**
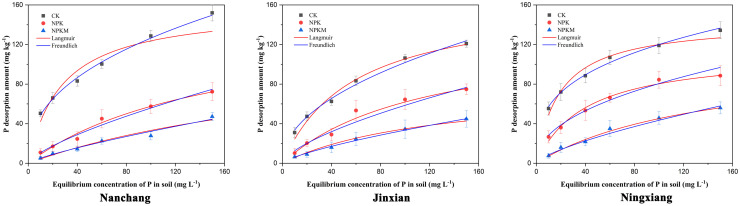
The desorption isotherms of P in red paddy soils under three different long-term experimental sites.

There is an error in the caption for Fig 2. Please see the complete, correct Fig 2 caption here.

The publisher apologizes for the errors.

## References

[1] Ahmed W, Jing H, Kailou L, Ali S, Tianfu H, Geng S, et al. (2021) Impacts of long-term inorganic and organic fertilization on phosphorus adsorption and desorption characteristics in red paddies in southern China. PLoS ONE 16(1): e0246428. 10.1371/journal.pone.0246428 33513183 PMC7846021

